# Wedge and needle liver biopsies show discordant histopathology in morbidly obese patients undergoing Roux-en-Y gastric bypass surgery

**DOI:** 10.1093/gastro/got006

**Published:** 2013-03-08

**Authors:** Sekou R. Rawlins, Cynthia M. Mullen, Howard M. Simon, Taewan Kim, Steven K. Landas, Marguerite S. Walser, Robert A. Levine

**Affiliations:** ^1^Division of Gastroenterology, Department of Medicine, State University of New York (SUNY), Upstate Medical University, Syracuse, NY, USA, ^2^Associates in Gastroenterology, Rockville, MD, USA, ^3^Department of Surgery, SUNY, Upstate Medical University, Syracuse, NY, USA, ^4^Department of Pathology, SUNY, Upstate Medical University, Syracuse, NY, USA and ^5^Section of Gastroenterology, Department of Medicine, Boston University School of Medicine, Boston, MA, USA

**Keywords:** gastric bypass, liver biopsy, steatosis, fibrosis image analysis

## Abstract

**Background:** Controversy exists over whether or not single-needle liver biopsies are sufficient to compare histological parameters in patients with non-alcoholic fatty liver disease.

**Aims:** To identify sampling variability, we biopsied four liver specimens per patient, based on biopsy size (needle vs wedge) and location (left vs right lobe), immediately prior to bariatric Roux-en-Y gastric bypass surgery.

**Methods:** Ten prospectively enrolled, morbidly obese patients underwent 40 laparoscopy-guided biopsies; two needle and two wedge from each of 16 left and 16 right liver lobes. The Kappa coefficient for concordance compared histological parameters from left and right lobe needle- and wedge biopsies. Wedge biopsies were considered our ‘Gold Standard’.

**Results:** Each patient had two wedge- and two needle liver biopsies. Kappa concordance between all needle and wedge biopsies from right and left lobes showed variability. Wedge- and needle liver biopsies from contralateral lobes had higher concordance with each other, compared to ipsilateral needle/wedge biopsy pairs. Contralateral wedge pairs had higher concordance than contralateral needle/needle pairs. There were no biopsy complications.

**Conclusions:** Wedge biopsy pairs had the best Kappa concordance but contralateral needle/needle biopsy pairs had good Kappa concordance. There were no complications from the 40 needle- and wedge liver biopsies, confirming the safety of laporoscopic multi-biopsy in both liver lobes.

## INTRODUCTION

Despite advances in imaging and surrogate biomarkers, needle liver biopsy remains diagnostic for staging of liver diseases. There is debate concerning the incidence of needle liver biopsy sampling error, due to its limited liver sample size, 1/50 000 of the liver [1–2]. This controversy has been addressed previously in patients with chronic liver disease [3–5] and includes patients undergoing gastric bypass for weight loss, since non-alcoholic fatty liver disease (NAFLD) is present in approximately 90% [6–7] and cirrhosis in about 26% at the time of surgery [8–9]. In NAFLD, studies assessing sample variability employed multiple needle biopsies from either a single lobe or from both left and right lobes of the liver [10–13]. Consensus correlation of steatosis between two liver samples was generally good, while that for inflammation was only fair to moderate [10–13]. Severity of fibrosis had wide pathological variability between paired liver biopsy samples, ranging from 0–40% [10–13].

Studies have not been performed in obese individuals with NAFLD using paired needle vs wedge biopsies in both left and right liver lobes. A wedge biopsy provides a 40-fold larger histological sample than the most commonly used 16 gauge needle biopsy and 20-fold greater sample size than a 14 gauge needle biopsy [14]. Thus, wedge biopsy is likely to be more representative of the liver parenchyma as a whole. Smaller standard liver biopsies using 14- or 16 gauge needles may result in increased sample variability and thereby underestimate the extent of fibrosis [14–16].

Our aim was to determine clinical significance to detect histopathology with one or more liver biopsies, how many biopsies were needed and the safety of the procedure in pre-operative gastric bypass patients. We evaluated for the first time results of paired wedge- and needle biopsies from both hepatic lobes obtained from the same patients.

## MATERIALS AND METHODS

### Study Population

Twenty-one morbidly obese patients undergoing bariatric Roux-en-Y gastric bypass surgery at State University of New York Upstate Medical University were prospectively enrolled between September 2009 and June 2010. The protocol was approved by the Institutional Review Board at SUNY Upstate Medical University and all patients provided written, informed consent. Patients were excluded if they consumed >20 g of alcohol daily, relayed a history of significant alcohol use in the past, hepatitis B and C, iron overload or other chronic, documented liver disease. Standard clinical, anthropometric and laboratory information were obtained. Demographics and baseline laboratory characteristics of the biopsied patient cohort are summarized in Table 1.

**Table 1.** Baseline biopsied patient characteristics (*n* = 10**)**

Variable	Value
Age	33.5 (23–42)
Female	75%
Hypertension	60%
Diabetes/glucose intolerante	50%
Metabolic syndrome	50%
BMI, median (kg/m^2^)	45 (37–56)
Aspartate aminotransferase (U/L)	22 (14–50)
Alanine aminotransferase (U/L)	27 (19–83)
Total bilirubin (mg/dl)	0.3 (0.2–0.6)
Random glucose	92 (81–135)

Note: values expressed for percentages; value range expressed in parentheses.

Immediately prior to the bypass procedure, each patient underwent a laparoscopically guided needle biopsy, using one pass with a 16-gauge Tru-cut biopsy instrument (Cardinal Health, Ohio) and a standard 1 × 1 cm wedge biopsy from each lobe of the liver under laparoscopic guidance, obtained via scalpel dissection followed by harmonic tool (Endoshears) for hemostasis. Biopsies were performed prior to the surgery to minimize the risk of ‘surgical hepatitis’ that could affect the amount of inflammation seen in such biopsies. Needle biopsy size was adequate: at least 1.5 cm in length with ≥5 portal zones.

### Histological methods

Histopathology by semiquantitative assessments was evaluated according to the system established by the Clinical Research Network for non-alcoholic steatohepatitis, as published by Kleiner *et al.* [17]. Briefly, the NAFLD activity score is the unweighted sum of the scores for steatosis (0–3), lobular inflammation (0–3) and ballooning (0–2); thus ranging from 0–8. Steatosis was graded 0–3 as follows: 0 = <5% steatosis; 1 = 5–33%; 2 = 33–66% and grade 3 = >66%. Lobular inflammation was scored based on the number of inflammatory foci per 200x per field as follows: 0 = no inflammatory foci; 1 = <2 foci; 2 = 2–4 foci and 3 = >4 foci. Ballooning was scored as follows: 0 = none; 1 = few balloon cells present and 2 = prominent ballooning. NAFLD score <3 denoted ‘no NAFLD score’, 3–5 denoted ‘possible NAFLD score’ and ≥5 denoted ‘definite NAFLD score’. Fibrosis was staged 0–4 as follows: 0 = no fibrosis; 1 = periportal or perisinusoidal; 1A = mild, Zone 3, perisinusoidal; 1B = moderate, Zone 3, perisinusoidal; 1C = portal/periportal; 2 = perisinusoidal and portal/periportal; 3 = bridging fibrosis and 4 = cirrhosis.

### Semiquantitative analysis

Biopsy specimens were formalin-fixated and paraffin-embedded. Hematoxylin-eosin and Klatskin (Masson) trichrome stains were used to assess histopathology in each sample. A single experienced hepatopathologist (S.L.), blinded both to the patient identification and site of liver, semiquantitatively evaluated all biopsies. Adequate normal controls were confirmed for each batch of stained slides. For all of the biopsies, only tissue deeper than 2 mm from the capsule was assessed, in order to exclude capsule artifact. We considered the wedge biopsy to be our ‘Gold Standard’ and we compared needle biopsy with it for right and left liver lobes. Since only one of our patients had cirrhosis, our study was insufficient to evaluate differences in patients with cirrhosis.

To ensure no significant intra-observer variability, all 32 specimens were randomly selected and reviewed by the same pathologist and then, after a 4-month interval, for a second observation, re-blinded to the patient identity and biopsy site.

### Morphometric analysis

Digital quantification of fibrosis and digital quantification of steatosis were performed for each liver biopsy specimen, using the same trichrome-stained slides from the semiquantitative analysis, as described by Rawlins *et al.* [18]. Images were captured using a 40x magnification objective. These calculations used a system consisting of a binocular microscope attached to a 1300 x 1000 pixel resolution, color, digital camera (CoolSNAP-Pro*cf* color by Media Cybernetics, Silver Spring, MD, USA), a frame-grabber board installed in a Pentium PC and Image-Pro Plus 6.1 image analysis software (Media Cybernetics, Silver Spring, MD, USA). Simultaneous morphometric quantification of fibrosis and steatosis was performed from trichrome-stained liver slides as a percentage of the total sample. From the digital quantification of steatosis values, digital quantification of steatosis grade was derived using the system published by Kleiner *et al.* [17].

### Statistical analysis

Histopathologic agreement for steatosis, inflammation and fibrosis from semiquantitative analysis—as well as steatosis and fibrosis by morphometric analysis*,* between paired left and right lobe wedge biopsies, paired left and right lobe needle biopsies and wedge and needle biopsies from the same lobe—were assessed with Cohen’s Kappa coefficient of concordance [19]. This Kappa statistic assessed agreement for both histologic and morphometric analyses of steatosis grade and fibrosis stage between individual observations of left vs right and wedge vs needle techniques, wherein: Kappa <0.40 indicated poor agreement, 0.40–0.75 indicated fair-to-good agreement and >0.75 indicated excellent agreement. Analysis of variance with repeated measures was used to assess for differences amongst the four biopsy sites in digital quantification of steatosis and digital quantification of fibrosis [20].

## RESULTS

A total of 21 patients (20 Caucasian and 1 African-American), who had previously decided to undergo Roux-en-Y gastric bypass procedure for weight loss, were enrolled in the study at the pre-operative visit. At surgery, 11 of the enrolled patients did not receive liver biopsies, either due to patient preference or at the discretion of the operating surgeon at the time of the gastric bypass procedure. Ten other patients had needle- and wedge biopsies obtained from right and left lobes of the liver. There were no biopsy-related complications in the 10 patients that underwent the procedure and having 40 separate biopsies. Two of these patients’ biopsy samples were subsequently excluded because they were sent to the laboratory in saline instead of formalin and the pathologist (S.L.) believed this could create aberrations in histological analysis. Thus only eight patients (all Caucasian) had 32 biopsy samples for histological analysis. The clinical and laboratory data are presented in Table 1.

Characteristics of the eight biopsy samples and histological results are summarized on Table 2. Only one of eight patients had documented cirrhosis; the rest had varying degrees of minimal fibrosis. The average numbers of portal tracts per needle and wedge biopsy were 9.5 and 77.5, respectively. Steatosis was present in 62.5% of left needle-, 62.5% of right needle-, 75% of left wedge- and 50% of right wedge biopsies. Inflammation was present in 62.5% of left needle-, 75% of right needle-, 75% of left wedge- and 37.5% of right wedge biopsies. Fibrosis was present in 50% of left needle-, 62.5% of right needle-, 87.5% of left wedge- and 87.5% of right wedge biopsies. NAS was ≥5 in 12.5% of left needle-, 12.5% of right needle-, 25% of left wedge- and 12.5% of right wedge biopsies.

**Table 2.** Liver biopsy histopathology

	Needle biopsy	Needle biopsy	Wedge biopsy	Wedge biopsy
	Left lobe	Right lobe	Left lobe	Right lobe
	*n* = 8	*n* = 8	*n* = 8	*n* = 8
Portal Tracts (*n*), median	4.5	10	77	78
**Steatosis**				
0	3	3	2	4
1	3	2	4	1
2	2	3	1	2
3	0	0	1	1
**Lobular inflammation**				
0	3	2	2	5
1	4	4	3	2
2	1	3	3	1
3	0	0	0	0
**Ballooning**				
0	2	2	1	1
1	4	4	4	4
2	2	2	3	3
**Kleiner fibrosis stage [17]**				
0	4	3	1	1
1	2	3	3	3
1A	0	0	1	1
1B	0	0	0	0
1C	0	0	0	0
2	0	0	0	0
3	1	1	2	2
**Diagnostic classification (NAS)**				
Not steatohepatitis	3	3	2	4
Possible/borderline	2	4	3	3
Definite steatohepatitis	3	1	3	1

### Sampling variability

The level of agreement between wedge and needle biopsy samples is represented in Table 3. For fibrosis, as determined by the Kleiner system for non-alcoholic steatosis (NAS), concordance was fair-to-good to excellent, except between left needle and left lobe wedge biopsies, where it was found to be poor. Concordance was found to be fair-to-good for NAS, except between right needle/wedge biopsy pairs. For steatosis grade, concordance was fair-to-good, but poor between left needle/wedge biopsy pairs. For ballooning, concordance was good-to-excellent for all biopsies. For inflammation, concordance was fair-to-good between left vs right lobe needle biopsies and needle/wedge biopsy pairs.

**Table 3.** Concordance of biopsy samples

		Kappa coefficient (95% CI)		
	Left vs right	Left needle	Right needle	Left vs right
	needle biopsy	vs wedge biopsy	vs wedge biopsy	wedge biopsy
	*n* = 16	*n* = 16	*n* = 16	*n* = 16
**Steatosis grade**	0.62	0.27	0.47	0.51
				
**Lobular inflammation (% with)**	0.60	0.44	0.27	0.29
				
**Ballooning**	0.62	0.44	0.79	0.60
				
**Kleiner fibrosis stage**	0.46	0.15	0.48	0.82
				
**Ishak fibrosis stage**	0.40	0.22	0.65	0.66
				
**NAS**	0.46	0.22	0.65	0.66
				
**DQS grade**	0.57	0.20	0.44	0.46

DQS = digital quantification of steatosis.

Digital quantification of fibrosis % is a continuous variable and was not well-suited to Kappa concordance testing. Nevertheless, digital quantification of steatosis grades showed fair-to-good concordance between left and right lobes [Kappa concordance = 0.50, *P* < 0.01 (95% Confidence Interval {CI} 0.16–0.84)], between left and right wedge-, left and right needle- and right needle and right wedge biopsies. There was poor concordance between wedge and needle biopsies of the same side [Kappa concordance = 0.30, *P* = 0.09 (95% CI 0.05–0.66)].

Descriptive statistics for both digital quantification of steatosis % and digital quantification of fibrosis % are provided in Table 4. Using repeated measure analysis of variance there was no statistical difference between the four biopsy sites for digital quantification of steatosis % (*P* = 0.32) and digital quantification of fibrosis % (*P* = 0.17), given that the power to detect differences was small, 0.28 and 0.32, respectively, because of the small sample size.

**Table 4.** DQS and DQF descriptive statistics

	Mean DQS	Std. deviation	Mean DQF	Std. deviation
**Left wedge**	7.0	8.1	5.6	3.2
**Right wedge**	4.7	6.1	9.7	9.4
**Left needle**	7.1	7.5	5.7	5.3
**Right needle**	6.8	4.6	11.6	1.2

DQS = digital quantification of steatosis; DQF = digital quantification of fibrosis.

### Intra-obsterver concordance

Analysis of intra-obsterver agreement was calculated for all 32 individual specimens and identical, as summarized in Table 5, both before and after re-blinding. The grading of steatosis, staging of fibrosis and NAS had excellent concordance. Inflammation and staging of fibrosis by the Kleiner system had fair-to-good concordance between samples. Both ballooning and lobular inflammation had fair-to-good concordance.

**Table 5.** Intra-observer concordance

	Kappa coefficient
	(95% CI)
**Steatosis grade**	1.00
**Lobular inflammation (% with)**	0.47
**Ballooning**	0.47
**Kleiner fibrosis stage [17]**	0.62
**Non-alcoholic steatosis**	1.00

## DISCUSSION

Despite our small sample size, we found significant concordance statistics between needle- and wedge liver biopsies. Although safety was not the aim of our study, the safety of the multiple biopsy procedures is not surprising. We also observed as high as 75% steatosis in our biopsies, including one patient with cirrhosis, while patients with NAFLD and gastric bypass often have 90% steatosis. It is possible that our study patients may have been a healthier group than some of the 11 other patients who did not give consent for liver biopsy, or for whom the surgeons opted not to biopsy for various reasons.

The major finding in our study, with regard to histological and morphometric measurements, is generally strong concordance for all parameters (Table 3). The majority of Kappa values <0.4 (i.e. poor agreement) occurred in the comparison of wedge biopsies to the needle biopsies from right and left liver lobes; this despite the fact that Kappa values were almost always >0.4 in the comparisons of wedge biopsies and needle biopsies from both individual liver lobes, indicating inherent discordance in the interpretation of wedge and needle liver biopsies from the same or contralateral sides. We opted not to compare parameters from contralateral sides taken from different biopsy modalities, i.e. left needle vs right wedge or right needle vs left wedge, as we had reason to expect concordances would be similar or worse, compared to those observed for contralateral needle/needle or wedge/wedge pairs. We recognize that wedge biopsies are only available and easily procured during abdominal surgery, thus such is not practical clinically, but only as an opportunity to test the variability.

We observed a wide range of agreement of the level of fibrosis, from excellent (Kappa = 0.82) to poor (Kappa = 0.15) between wedge and needle biopsies. Previous studies noted discordance of at least one fibrosis stage in 12–41% of patients [10–13]. Surprisingly, Larson *et al.* challenged the notion that there may be marked variability in fibrosis in NAFLD by demonstrating concordance that was categorized as excellent (Kappa = 0.96) [10]. However, the most likely explanation for the robust findings of Larson *et al.* was larger sample area [10]; using a 14-gauge needle with biopsies 2–8 mm longer in size, contrasting to our and other studies [11–13], in which the more commonly-utilized 16-gauge needle was employed. Wedge biopsies provided more than a 10-fold increase in the number of portal tracts seen, even after deleting the areas of subcapsular collagen, the latter being a known histological artifact present in either needle or wedge biopsies [14–16, 21].

For steatosis grade we found good agreement between needle/needle biopsy pairs (Kappa = 0.62*)* and wedge/wedge biopsy pairs (Kappa = 0.51*).* In previous studies concordance of steatosis grade was good to excellent (Kappa = 0.64–0.91) [10–13]. We observed good-to-excellent concordance for ballooning; Kappa ranged from 0.44–0.79. Inflammation varied from poor to good; Kappa ranged from 0.27–0.60. Concordance regarding inflammation and ballooning varied widely among the aforementioned studies; K = 0.18–0.58 and Kappa = 0.2–0.73, respectively [10–13]. We found, for most parameters, that needle/needle and wedge/wedge biopsy pairs had superior concordance compared to ipsilateral wedge/needle biopsy pairs.

Although our morbidly obese patients underwent gastric bypass, we believe the results of this study may be generally applied to all patients with steatotic liver disease, as others have documented similar morphologic features of steatohepatitis in adults, regardless of the underlying etiology [22–25]. In addition, since steatosis due to excessive alcohol intake is often morphologically indistinguishable from non-alcoholic fatty liver disease, it is logical that these findings could be equally applicable to non-obese patients. Our findings may also be relevant for other chronic liver diseases, even though the typical Zone 3 hepatocellular injury of NAFLD differs from the portal-based inflammation, fibrosis and spotty lobular necrosis, as seen in other conditions, including hepatitis C [23–24].

The Kleiner system specifically assesses Zone 3 injury and reliably estimates sampling variability of portal fibrosis in morbidly obese patients [17]. Such findings could well apply to some degree in other chronic liver diseases, given their similar distributions.

The major limitation of our study was small sample size. However, in other large studies of paired liver biopsies with gastric bypass patients [10–13], there were greater numbers of subjects but having only two—not four—comparable data points. Because four biopsies were obtained from each of our eight patients, we were able to perform a sum of 32 liver biopsy analyses for the total group, which is numerically comparable to the number of samples in many of the prior published studies [10–13].

Prior studies in both hepatitis C patients and a heterogeneous patient population [3, 26], also showed significant histological differences between right and left liver lobes, further suggesting such variance beyond NAFLD. Our concordance data with the ‘Gold Standard’ wedge biopsy suggest that a minimum of two passes should be made at the time of every single-needle liver biopsy. We recognize that, compared to a single liver biopsy, multiple needle liver biopsies, even up to three biopsies [27], may increase histological concordance—but also risk.

The data suggest the reliability of needle biopsy, despite the presumed superiority of the wedge biopsy to assess NAFLD. Wedge biopsy pairs had higher concordance than ipsilateral needle/wedge biopsy pairs and point to a methodological difference that may need to be repeated in larger future studies. The data make clear the safety of multiple needle and wedge liver biopsies at the time of laparoscopic bariatric surgery.

**Conflict of interest:** none declared.
